# Long-Term Effects of Irrigation with Waste Water on Soil AM Fungi Diversity and Microbial Activities: The Implications for Agro-Ecosystem Resilience

**DOI:** 10.1371/journal.pone.0047680

**Published:** 2012-10-18

**Authors:** Maria del Mar Alguacil, Emma Torrecillas, Pilar Torres, Fuensanta García-Orenes, Antonio Roldán

**Affiliations:** 1 CSIC-Centro de Edafología y Biología Aplicada del Segura, Department of Soil and Water Conservation, Campus de Espinardo, Murcia (Spain); 2 Departamento de Biología Aplicada, Area de Botánica, Universidad Miguel Hernández. Avda, De la Universidad Elche, Alicante Spain; 3 Grupo de Edafología Ambiental, Departamento de Agroquímica y Medio Ambiente, Universidad Miguel Hernández, Alicante, Spain; Nanjing Agricultural University, China

## Abstract

The effects of irrigation with treated urban wastewater (WW) on the arbuscular mycorrhizal fungi (AMF) diversity and soil microbial activities were assayed on a long-term basis in a semiarid orange-tree orchard. After 43 years, the soil irrigated with fresh water (FW) had higher AMF diversity than soils irrigated with WW. Microbial activities were significantly higher in the soils irrigated with WW than in those irrigated with FW. Therefore, as no negative effects were observed on crop vitality and productivity, it seems that the ecosystem resilience gave rise to the selection of AMF species better able to thrive in soils with higher microbial activity and, thus, to higher soil fertility.

## Introduction

In arid and semiarid areas around the world water shortage is one of the most serious environmental problems, which necessitates the search for alternative sources of good-quality water to satisfy this demand. The use of treated wastewater for irrigation is one of the most-readily-available alternative water sources when natural resources are scarce. This water reuse in agriculture is increasing in many places around the world [1], [2] and the implications for the sustainability of agroecosystems deserve careful attention.

Arbuscular mycorrhizal fungi (AMF) form mutualistic associations with most land plants. These fungi provide beneficial effects to plants, increasing their growth, uptake of nutrients, principally phosphorous and protection against biological and environmental stresses. The fungi in return receive plant carbon assimilates or carbohydrates [3]. It is evident that AMF play an important role in the edaphic system, contributing to the maintenance of terrestrial ecosystem functioning. Also, their occurrence, activity and efficency can be valuable indicators of soil quality [4]. It has been shown that the diversity of AMF in the soil can affect both productivity and ecosystem functioning [5], [6]. Therefore, knowledge of the diversity of the AMF in the soil of crops is essential for better management, sustainability and productivity of these agricultural soils.

In spite of the important role that AMF play in the agricultural ecosystems, seems to be no information on the effect of treated urban wastewater on AMF diversity. Only, Ortega-Larrocea et al., [7] determined the abundance of AMF morphotypes in soil irrigated with untreated urban wastewater in central Mexico, however the results cannot be comparable since the untreated wastewater can incorporate significant amounts of pollutants, such as heavy metals in the soil that can depress the AMF colonization [8].

The objective of this study was to assess the effect of the long-term irrigation with treated urban wastewater on the AMF diversity in a field of *Citrus* sp. trees. Also, we determined whether the irrigation with treated wastewater had any effect on soil quality parameters related to soil microbial activity.

## Results and Discussion

**Figure 1:**
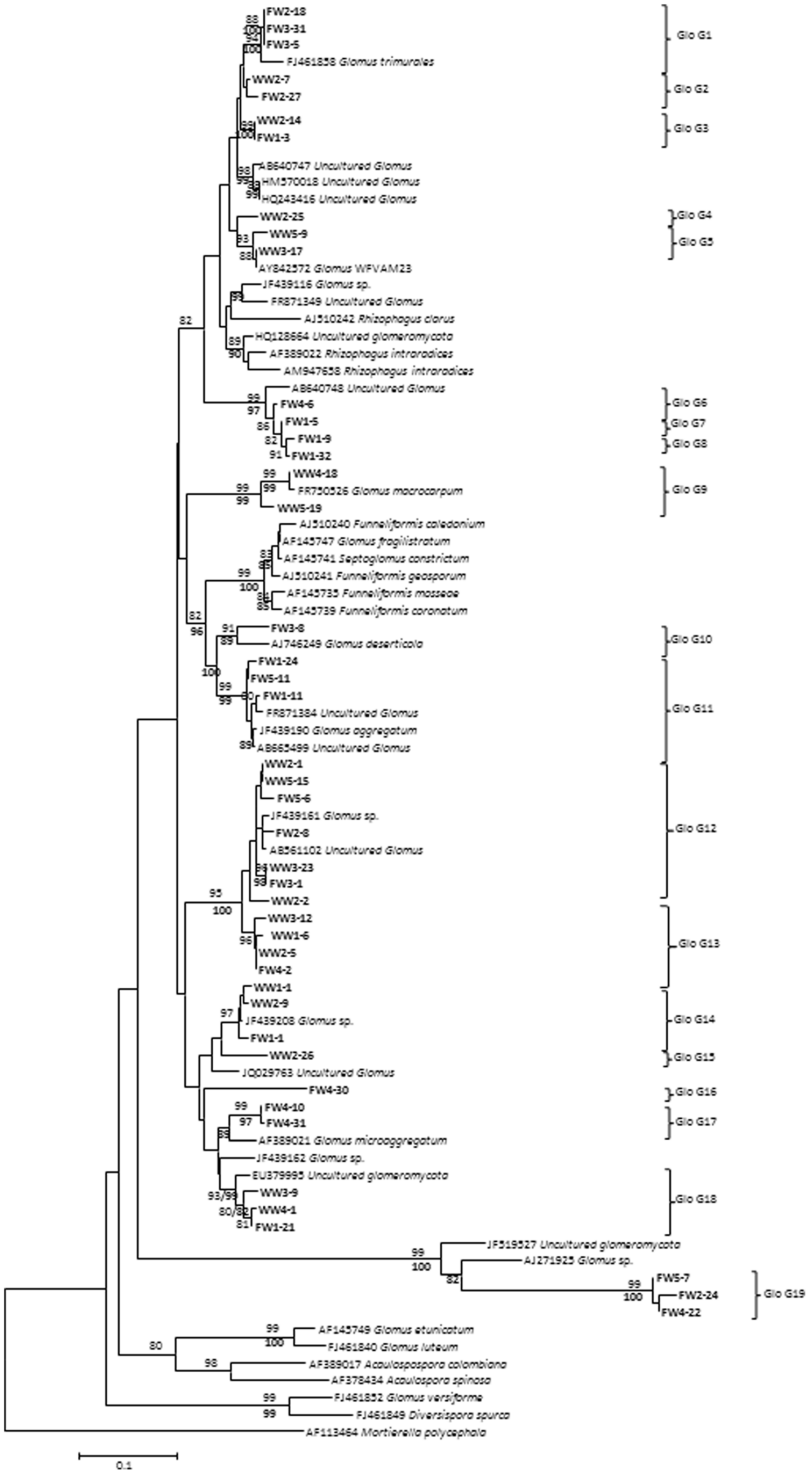
Neighbour-Joining (NJ) phylogenetic tree showing AMF sequences isolated from soil irrigated with freshwater (FW) and treated wastewater (WW) and reference sequences from GeneBank. Numbers above the branches indicate the bootstrap values (above 80%, 1000 replicates) of the NJ analysis; numbers below the branches indicate the bootstrap values of the maximum likelihood analysis. Sequences obtained in the present study are shown in bold type. *Mortierella polycephala* was used as out-group.

In this study, 320 clones from 10 clone libraries (five repetitions per treatment) were screened by PCR; out of these, 170 clones containing an LSU rDNA fragment were sequenced and 145 sequences of AM fungal origin were grouped in 19 AMF sequence types, all belonging to the family Glomeraceae and with similarities varying from 97 to 100% and bootstrap values ≥80% (Fig. 1).

**Figure 2:**
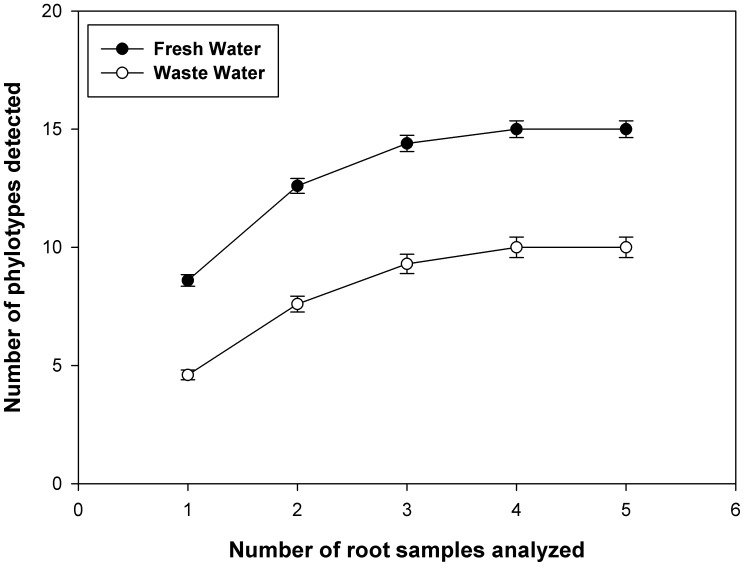
Sampling effort curve for the AM fungal sequence types richness observed in this study. The sample order was randomized by 100 replications in EstimateS, version 8.0 [32]. Bars represent error standard (n = 5).

The sampling effort curves (Fig. 2) indicated that the number of sequences analysed per sample was sufficient to provide coverage of the AM fungal diversity in the soils irrigated with WW and FW, since both curves reached the plateau. Four AMF sequence types were related to sequences from morphologically-described species (Glo G1 to *Glomus trimulares*, Glo G9 to *Glomus macrocarpum,* Glo G10 to *Glomus deserticola* and Glo G11 to *Glomus aggregatum*). The rest of the AMF sequence types were either related to uncultured glomalean species or were not related to any sequences of AMF in the database (Fig.1).

The soil irrigated with FW had a higher number of AMF sequence types (15) and higher AMF diversity index (*H* ´ = 2.40) than the soil irrigated with WW (*H* ´ = 1.94 and 10 AMF sequence types). Thus, the influence of the type of water was statistically significant (*P*  = 0.040). The lower AMF diversity found in the soil irrigated with WW could be attributed to an additional input of nutrients in the wastewater (Table 1); in fact, there is evidence of reduced AMF diversity and shifts in community composition in nutrient-enriched areas [9], [10]. Some studies have pointed out that changes in soil properties are the factors influencing the structure of AMF communities in agricultural systems [11], [12], [13], [14], [15] due to the different requirements of the fungi for C, N or P [16]. We found that all enzymatic activities measured (alkaline phosphatase, urease, dehydrogenase, protease and β-glucosidase) were significantly higher in the soils irrigated with WW than in the soils irrigated with FW (Table 2). Also, the exchangeable P was significantly higher in the soil irrigated with WW. An enhancement of the soil enzyme activities after 10 and 20 years of irrigation with treated municipal wastewater was observed by Chen et al. [17] and Adrover et al. [18], respectively, in accordance with our results. The recorded beneficial effect of WW irrigation on soil microbial biomass and the related activities can be attributed to the addition of easily-decomposable organic matter and nutrients [17], [19]. Moreover, BOD/COD values higher than 0.1 in wastewater mean that it is classified as biodegradable organic matter [20]. Thus, the organic matter of WW in our study can be decomposed easily by microorganisms in the soil, since the BOD/COD ratio was 0.2 (Table 1).

**Table 1:** Characteristic of the treated wastewater (WW) and fresh water (FW) used for the irrigation.

Parameters	Average of WW	Average of FW
pH (u.pH)	8.1	7.72
EC (µS/cm)	2620	1760
SS (mg l^−1^)	15	2
BOD_5_(mgO_2_ l^−1^)	7	<1
COD(mgO_2_ l^−1^)	31.9	4.2
Nk (mg l^−1^)	43	<1
Ca^2+^ (mg l^−1^)	122	80
Na^+^ (mg l^−1^)	270	130
K^+^ (mg l^−1^)	19.9	2.7
Mg^2+^ (mg l^−1^)	41	40
B (mg l^−1^)	0.5	<0.01
P (mg l^−1^)	2.48	<0.01
Fe (mg l^−1^)	0.071	<0.01
Mn (mg l^−1^)	0.019	<0.01
Zn (mg l^−1^)	0.012	<0.01
Cl^-^(mg l^−1^)	453	280
SO4^2−^(mg l^−1^)	316	130
NO_3_^−^ (mg l^−1^)	0.22	<0.01

BOD_5_: biochemical oxygen demand; COD: Chemical Oxygen Demand; SS: suspended solids; Nk: Nitrogen Kjeldahl.

**Table 2:** Biological and physico-chemical properties of rhizosphere soil of *Citrus sp.* irrigated with fresh water (FW) or treated wastewater (WW) 43 years after establishment (n = 5).

	FW	WW	Significance
Alkaline Phosphatase	1.51±0.05	3.99±0.20	*P*<0.05
(µmol PNP g^−1^ h^−1^)			
Urease	0.42±0.03	1.29±0.08	*P*<0.05
(µmol NH_3_ g^−1^ h^−1^)			
Dehydrogenase	89.90±1.80	106.95±2.53	*P*<0.05
(µg INTF g^−1^ soil)			
Protease	2.48±0.10	3.67±0.15	*P*<0.05
(µmol NH_3_ g^−1^ h^−1^)			
β−γλυχοσιδασε	1.71±0.10	3.82±0.23	*P*<0.05
(µmol PNP g^−1^ h^−1^)			
Exchangeable P (mg kg^−1^)	112.7±1.5	137.1±1.7	*P*<0.05
EC (µS cm^−1^)	412±28	496±83	n.s.
pH	8.3±0.1	7.9±0.1	n.s.
TOC (g kg^−1^)	22±4	30±2	*P*<0.05

Mean ± SE. n.s.; not significant. TOC: total organic carbon.

These results are also supported by a significant, negative correlation between the biodiversity of the AMF, measured as the Shannon index, and the dehydrogenase (r = −0.657; P<0.05), urease (r = −0.839; P<0.01), protease (r = −0.719; P<0.05), alkaline phosphatase (r = −0.881; P<0.01) and β-glucosidase (r = −0.803; P<0.01) activities, as well as the exchangeable P in the soil (r = −0.862; P<0.01). This confirms that the AMF diversity appears to vary in response to the soil quality parameters in field studies [10].

**Figure 3:**
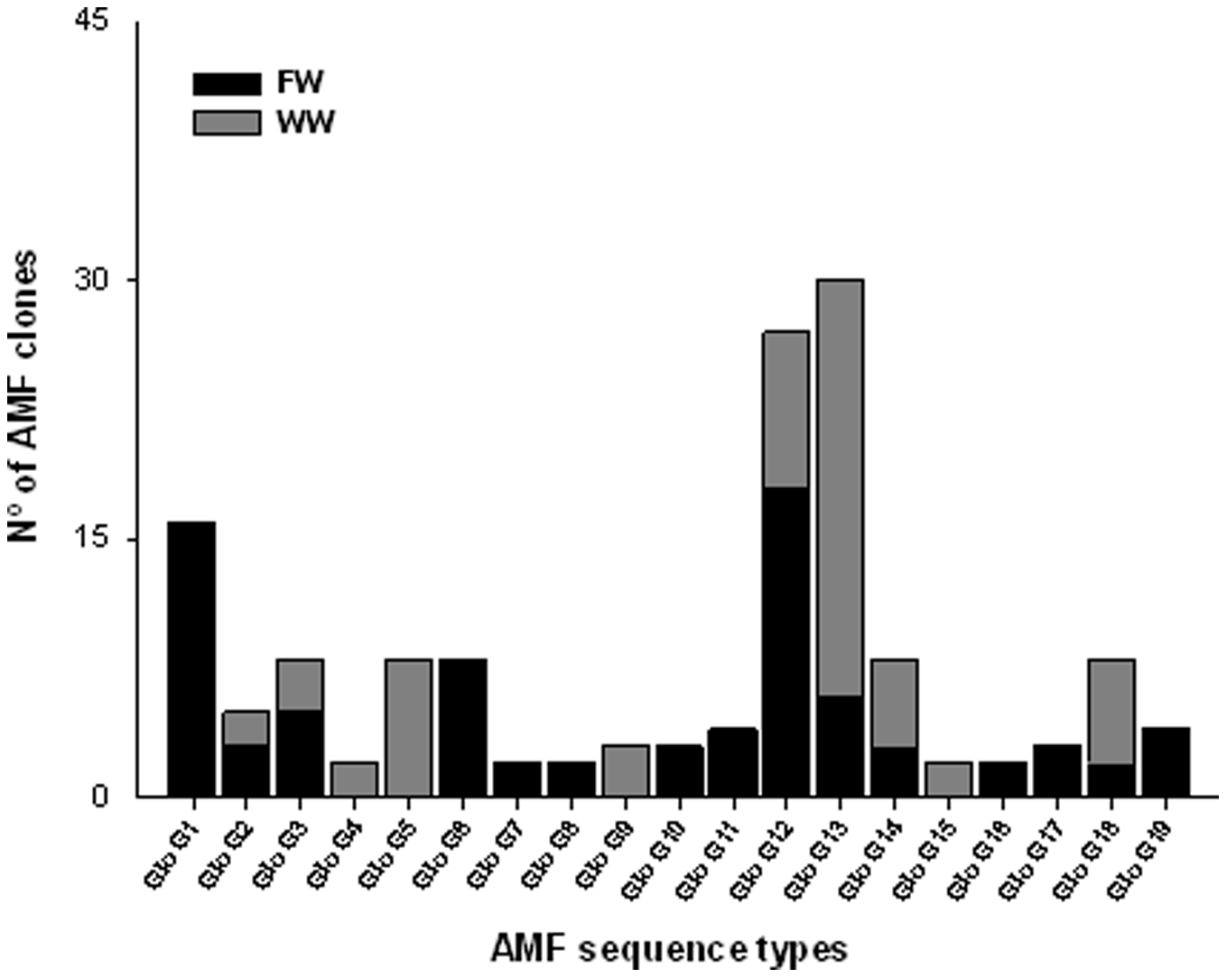
Bar plot showing the number of clones detected for each AM fungal sequence type in the soil irrigated with fresh water (FW) and treated wastewater (WW) 43 years after establishment.

**Figure 4:**
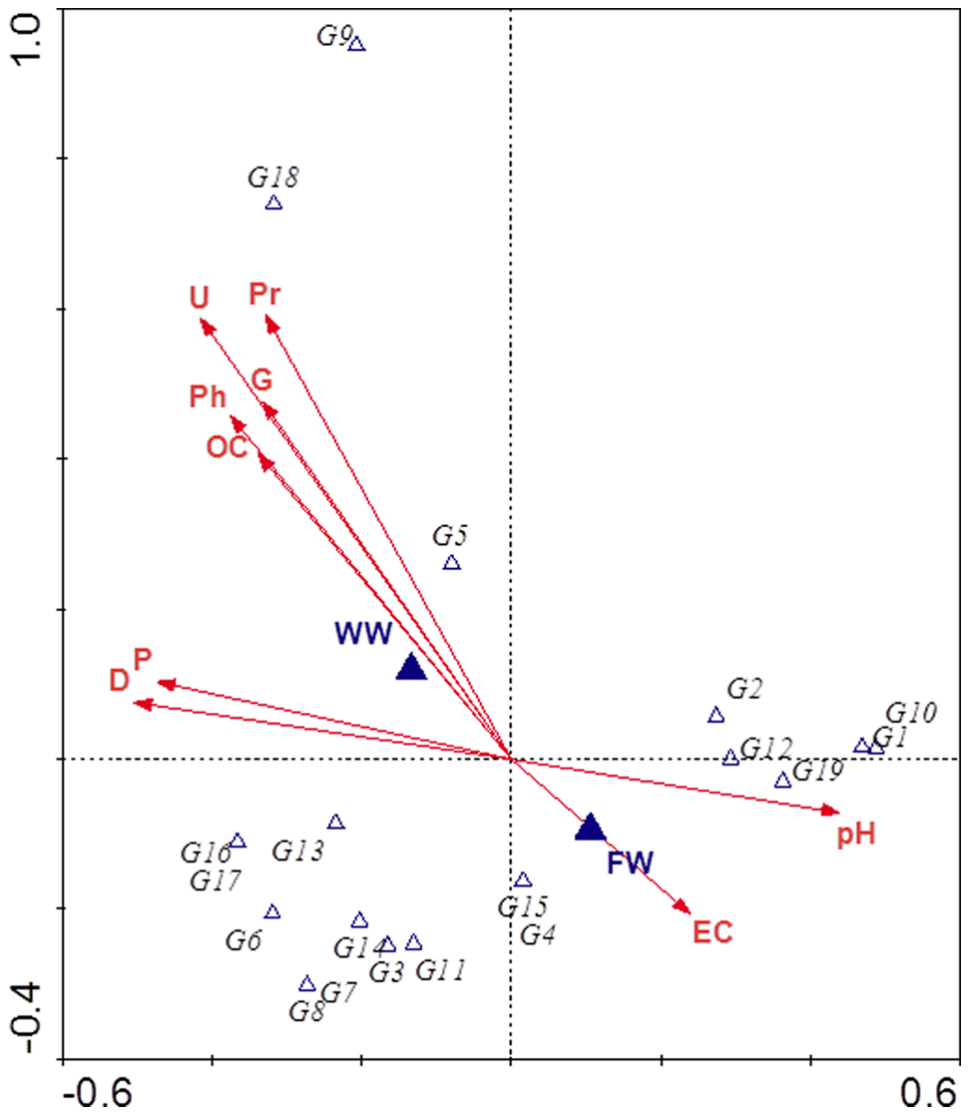
Canonical correspondence analysis (CCA) of the AMF communities found and soil properties at the soil irrigated with fresh water (FW) and treated wastewater (WW). The eigenvalues of the first and second axes in the two-dimensional ordination diagrams are as follows: CCA1∶0.82 and CCA2∶0.65. Ph: Alkaline Phosphatase, U: Urease, D: Dehydrogenase, Pr: Protease, G: β-glucosidase, OC: Total organic carbon, EC: Electrical conductivity, P: exchangeable P.

There were higher number of AMF sequence types occurring exclusively in the soils irrigated with FW (GloG1, GloG6, GloG7, GloG8, GloG10, GloG11, GloG16, GloG17 and GloG19) than in those irrigated with WW (GloG4, GloG5, GloG9 and GloG15) (Fig. 3). This difference in the AMF community composition between WW and FW treatments was shown also in the CCA diagram (Fig. 4). The rest of the AMF sequence types (GloG2, GloG3, GloG12, GloG13, GloG14 and GloG18) were found in soils irrigated with either type of water. This change in the AMF community composition could have been due to selection of the types of AMF better able to proliferate and survive under the prevailing soil conditions [10]. In this sense, positive, significant correlations were observed between the urease (r = 0.642; P<0.05) and protease (r = 0.688; P<0.05) activities and the GloG18 fungal type as well as between the exchangeable P (r = 0.672; P<0.05) and GloG13 (Table 3), both AMF types appearing in the highest proportion when the soil was irrigated with WW (Fig. 3). These results were clearly represented in the CCA analysis (Fig. 4).Since changes in enzyme activities are mediated by shifts in microbial populations; it can be assumed that the reorganization of the edaphic microbial communities both in function and composition could exert a clear influence on AMF communities.

**Table 3:** Pearson’s coefficients of correlation and significance level between the soil parameters measured, and the AMF community composition

	G1	G2	G3	G4	G5	G6	G7	G8	G9	G10	G11	G12	G13	G14	G15	G16	G17	G18	G19
**Dehydrogenase**	-0.508	-0.159	0.131	0.563	0.46	-0.016	-0.193	-0.193	0.071	-0.375	-0.304	-0.27	0.394	-0.086	0.563	0.172	0.172	0.196	-0.396
	-0.134	-0.662	-0.719	-0.09	-0.181	-0.966	-0.594	-0.594	-0.846	-0.285	-0.394	-0.451	-0.26	-0.812	-0.09	-0.635	-0.635	-0.588	-0.257
**Urease**	-0.441	-0.111	-0.171	0.113	0.587	-0.361	-0.258	-0.258	0.617	-0.395	-0.375	-0.392	0.157	-0.138	0.113	-0.224	-0.224	0.642*	-0.405
	-0.202	-0.761	-0.636	-0.755	-0.074	-0.305	-0.472	-0.472	-0.057	-0.259	-0.286	-0.262	-0.664	-0.705	-0.755	-0.534	-0.534	-0.045	-0.246
**Protease**	-0.342	0.124	-0.256	0.051	0.368	-0.399	-0.315	-0.315	0.58	-0.409	-0.429	-0.343	0.216	-0.121	0.051	-0.217	-0.217	0.688*	-0.165
	-0.333	-0.733	-0.475	-0.888	-0.295	-0.254	-0.376	-0.376	-0.079	-0.24	-0.216	-0.331	-0.548	-0.739	-0.888	-0.547	-0.547	-0.028	-0.648
**Alkaline Phosphatase**	-0.427	-0.201	-0.255	0.168	0.579	-0.474	-0.383	-0.383	0.433	-0.33	-0.457	-0.351	0.39	-0.052	0.168	-0.249	-0.249	0.504	-0.46
	-0.219	-0.577	-0.477	-0.642	-0.079	-0.166	-0.274	-0.274	-0.211	-0.352	-0.184	-0.32	-0.265	-0.887	-0.642	-0.488	-0.488	-0.138	-0.181
β**−γλυχοσιδασε**	-0.378	-0.085	-0.348	0.081	0.479	-0.507	-0.435	-0.435	0.412	-0.346	-0.493	-0.346	0.43	-0.074	0.081	-0.242	-0.242	0.529	-0.304
	-0.281	-0.815	-0.324	-0.824	-0.162	-0.134	-0.209	-0.209	-0.237	-0.327	-0.148	-0.328	-0.214	-0.838	-0.824	-0.501	-0.501	-0.116	-0.393
**Exchangeable P**	-0.542	-0.387	-0.098	0.366	0.251	-0.401	-0.328	-0.328	0.206	-0.335	-0.339	-0.368	0.672*	-0.354	0.366	-0.207	-0.207	0.199	-0.583
	-0.106	-0.269	-0.788	-0.299	-0.484	-0.251	-0.355	-0.355	-0.569	-0.344	-0.339	-0.295	-0.033	-0.316	-0.299	-0.566	-0.566	-0.582	-0.077
**EC**	0.166	0.097	-0.246	-0.231	0.236	-0.286	-0.134	-0.134	-0.264	0.094	-0.175	0.112	0.333	0.189	-0.231	-0.248	-0.248	-0.603	-0.013
	-0.647	-0.79	-0.494	-0.52	-0.511	-0.423	-0.713	-0.713	-0.461	-0.795	-0.629	-0.757	-0.347	-0.6	-0.52	-0.49	-0.49	-0.065	-0.971
**pH**	0.253	0.284	0.248	-0.054	0.541	-0.081	0.307	0.307	-0.161	0.126	0.225	0.316	-0.455	-0.104	-0.054	-0.415	-0.415	-0.295	-0.016
	-0.481	-0.427	-0.49	-0.882	-0.106	-0.823	-0.388	-0.388	-0.657	-0.728	-0.533	-0.374	-0.187	-0.775	-0.882	-0.233	-0.233	-0.408	-0.964
**TOC**	-0.403	-0.316	-0.16	0.241	0.406	-0.482	-0.321	-0.321	0.603	-0.241	-0.421	-0.291	0.307	0.116	0.241	-0.321	-0.321	0.373	-0.654
	-0.248	-0.374	-0.66	-0.502	-0.244	-0.158	-0.365	-0.365	-0.065	-0.502	-0.226	-0.415	-0.389	-0.75	-0.502	-0.365	-0.365	-0.288	-0.05

In conclusion, the long-term irrigation of an orange-tree orchard with WW reduced the AMF diversity in the soil but, as no negative effects were observed on crop vitality and productivity, it seems that the ecosystem resilience resulted in the selection of AMF species better able to thrive in the soils with higher microbial activity and, thus, to improved soil fertility.

## Materials and Methods

### Ethics Statement

No specific permits were required for the described field studies since these locations are not privately-owned or protected in any way. Field studies did not involve endangered or protected species.

### Study Site and Experimental Design

This research was conducted in an area located in Alicante (Southeast Spain) (coordinates 38° 17′ 38″N, 0° 33′ 50″W). The soil of this study is classified as a Xerorthent [21]. For 43 years, an experimental *Citrus aurantium* L. (orange-tree) orchard has been drip-irrigated with water from an urban wastewater plant with secondary treatment by activated sludge, while control plots subjected to drip irrigation with freshwater were also established during all of the experimental period. The main characteristics of the two types of water used for the irrigation are shown in Table 1.

In June 2011, rhizosphere soil samples from individual trees were collected in a randomised design with five replicates for each irrigation treatment: irrigation with treated waste water (WW) and irrigation with fresh water (FW).

### Soil Biological, and Physico-chemical Analyses

Dehydrogenase, urease, N-α-benzoyl-L-argininamide (BAA) hydrolyzing protease, alkaline phosphatase and β-glucosidase activities were assayed following the procedure described in [22]. Soil pH and electrical conductivity were measured in a 1∶5 (w/v) aqueous solution. Exchangeable P, extracted with 0.5 M NaHCO_3_ (Panreac) was determined by colorimetry according to Murphy and Riley [23] and the total organic C was determined by oxidation with potassium dichromate in a sulphuric medium and excess dichromate evaluated using Mohr’s salt [24].

### Molecular Analysis

All PCR experiments were run using DNA preparations consisting of pooled soil extracts for each sample. DNA extractions from 10 soil samples were carried out. For each of the 10 soil samples, genomic DNA was extracted from 0.5 g of soil fresh weight using a FastDNA™ Spin kit for soil according to the recommendations of the manufacturer (Q-BIOgene, Heidelberg, Germany). Two-microliter samples of genomic DNA were used for the amplification of a partial large-subunit (LSU) rRNA gene region. In order to enhance the efficiency of the amplification and increase the amount of DNA available for cloning, a heminested PCR was carried out using the primer pairs LR1 [25] and FLR2 [26] for the first amplification step and LR1 and FLR4 [27] for the second one. PCR reactions were carried out in a final volume of 25 µl using the “ready to go” PCR beads (Amersham Pharmacia Biotech, Buckinghamshire, United Kingdom), and 0.5 µM of each primer (PCR conditions: 30 cycles at 93°C for 1 min, 58°C for 1 min, and 72°C for 1 min, followed by a final extension period at 72°C for 5 min). Two microlitres, from the first PCR, were used as template DNA in a second PCR reaction under the same PCR conditions. All the PCR reactions were run on a Perkin Elmer Cetus DNA Thermal Cycler. Reactions yields were estimated by using a 1.2% agarose gel containing *GelRed*™ (Biotium, Hayward, California). The PCR products were purified using a Gel extraction Kit (Qiagen) cloned into pGEM-T Easy (Promega, Madison, Wisconsin, USA) and transformed into Escherichia coli (XL2-Blue). Thirty two positive transformants were screened in each resulting LSU rRNA gene library, using 0.8 units of RedTaq DNA polymerase (Sigma-Aldrich, St. Louis, Missouri, USA) and a re-amplification with LR1 and FLR4 primers with the same conditions described above. All clones having inserts of the correct size in each library were sequenced. The sequencing was done by Laboratory of Sistemas Genómicos (Valencia, Spain).

Forty-four representative sequences of the clones generated in this study have been deposited at the (NCBI) GenBank (http://www.ncbi.nlm.nih.gov) under the accession numbers HE858374-HE858417.

### Phylogenetical Analysis

Phylogenetic analysis was carried out on the sequences obtained in this study and those corresponding to the closest matches from GenBank. Sequences were aligned with other published glomeralean sequences using the program BioEdit software [28]. Neighbour-joining (NJ) and maximum likelihood (ML) phylogenetic analyses were performed with the programs MEGA software v. 4 [29] and RAxML v.7.0.4 [30], respectively. Distances for the NJ tree were computed using the default parameters. For the ML analysis, a GTR-GAMMA model of evolution was used.

Different AMF sequence types or phylotypes, were defined as groups of closely related sequences, usually with a high level of bootstrap support in the phylogenetic analyses (higher than 85%) and sequence similarity ≥ 97%. The pairwise analysis within clusters was carried out using BioEdit software [28].

### Diversity of AM Fungal Community

The Shannon (H’) index was calculated as an additional measure of diversity, as it combines two components of diversity, i.e., species richness and evenness. It is calculated from the equation H’ =  - ∑*pi*(ln *pi*), where *pi* is the number of sequences belonging to each AM sequence type relative to the total number of sequences.

### Statistical Analysis

The data were subjected to analysis of variance, and comparisons among means were made using a Least Significant Difference (LSD) test calculated at *p*<0.05. Correlation analysis between all the soil parameters measured and the AMF diversity was carried out using Pearsońs rank correlation coefficients. Statistical procedures were carried out with the software package IBM SPSS Statistic 19.0 for Windows.

In order to investigate the effect of the long-term irrigation with treated urban wastewater on the AM fungal community composition and to correlate the AM fungal community composition with the soil properties related to soil microbial activity, ordination analyses were conducted in CANOCO for Windows v. 4.5 [31] using the relative abundance data for each AMF sequence type. Initial detrended correspondence analysis suggested a unimodal character of the data response to the sample origin (the lengths of gradients were >4); therefore, the canonical-correspondence analysis (CCA) was used. Monte Carlo permutation were conducted using 499 random permutations.

The number of clones of each AMF sequence types in each soil sample was used to construct the sampling effort curves (with 95% confidence intervals) using the software EstimateS 8.00 [32]. The sample order was randomized by 100 replications.
